# Deciphering the tomato phosphate starvation response: Functional and transcriptional roles of SlPHR3 and SlPHR4

**DOI:** 10.1093/plcell/koaf197

**Published:** 2025-08-09

**Authors:** Andrea Gómez-Felipe

**Affiliations:** Assistant Features Editor, The Plant Cell, American Society of Plant Biologists; Department of Biology, Indiana University, Bloomington, IN 47405, USA

Phosphorus (P) is an essential nutrient for plants, primarily taken up from the soil in the form of inorganic phosphate (Pi) (Paz-Ares et al. 2022). However, the fraction of soluble Pi available to roots represents only a small portion of the total phosphorus in the soil (Holford 1997; Prathap et al. 2022). Pi is relatively immobile due to its strong adsorption to soil particles and its tendency to form insoluble complexes with calcium, iron, and aluminum oxides or hydroxides, processes that are highly influenced by soil composition and pH (Poirier et al. 2022). The low mobility of Pi in soil poses a significant challenge for plant productivity (Bucher 2006).

To cope with Pi deficiency, plants activate a set of adaptive mechanisms collectively known as phosphate starvation responses (PSRs) (Bates and Lynch 1996; Chien et al. 2018). These responses are coordinated by key transcriptional regulators, such as PHOSPHATE STARVATION RESPONSE 1 (PHR1) in *Arabidopsis* and its orthologs in other species such as rice (Guo et al. 2015; Sun et al. 2016). Despite the advances made in model systems, there is still a lot to learn about the regulation of PSRs in other crops such as tomato (*Lycopersicon esculentum*). Tomato is an important crop with low P use efficiency, and its growth and yield are affected by Pi deficiency. Although genes associated with PSRs have been identified in tomato, the mechanisms governing Pi metabolism and PSRs in this crop remain elusive.

Addressing this knowledge gap, **Dongbo Lin and coauthors (Lin et al. 2025)** identified and characterized 2 PHR1-like transcription factors, SlPHR3 and SlPHR4, as central regulators of Pi homeostasis and PSRs. Using a combination of genetic, physiological, and transcriptomic approaches, the authors uncovered a phosphate-responsive regulatory network involving multiple downstream transcription factors and metabolic pathways, including anthocyanin biosynthesis.

To explore the physiological responses of tomato to Pi availability, the researchers grew 10-day-old seedlings hydroponically under Pi-sufficient (FPi) and Pi-deprived (NPi) conditions for 7 days. Half of the NPi-treated seedlings then received Pi resupply for 24 h. Under Pi deprivation, shoot biomass significantly declined after 5 days, while root biomass increased by day 7, resulting in a higher root-to-shoot ratio. Anthocyanin content rose from day 3 in NPi-treated plants. Both roots and shoots showed a gradual decrease in Pi concentrations during Pi starvation, with Pi resupply noticeably restoring Pi levels.

Analysis of Pi-sufficient and NPi-treated tomato seedlings revealed a cluster of Pi deficiency-induced genes in “PSR modules.” Notably, genes in these modules are enriched in the PHOSPHATE STARVATION RESPONSE1 binding site (P1BS) cis-regulatory motif, suggesting that PHR factors may govern the transcriptional network of PSRs in tomato. Among the tomato PHR genes, SlPHR3 and SlPHR4 (homologs of *Arabidopsis* PHR1/PHL1) had not been functionally characterized. To clarify their roles in PSR modulation, overexpression and CRISPR-Cas9 double knockout experiments were conducted. Overexpression lines (*SlPHR3-OE* and *SlPHR4-OE*) accumulated more Pi, exhibited increased biomass, and showed elevated anthocyanin levels under Pi starvation. In contrast, *slphr3 slphr4* double mutants displayed reduced Pi accumulation, biomass, and anthocyanin content. These results showed that both transcription factors act as positive, likely redundant, regulators of Pi homeostasis and PSRs in tomato.

To further elucidate the downstream regulatory network of SlPHR3 and SlPHR4, the authors investigated their potential direct targets, genes known to be involved in Pi metabolism. Using electrophoretic mobility shift assays, dual-luciferase reporter assays, and chromatin immunoprecipitation analyses, they showed that SlPHR3 and SlPHR4 directly bind to the promoters of target genes and activate their transcription, particularly under Pi-deprived conditions.

These findings provided a foundation for exploring additional target genes by focusing on transcription factors in the “PSR modules” as potential new targets of SlPHR3 and SlPHR4. Lin et al. identified 3 transcription factors as direct downstream targets of SlPHR3 and SlPHR4: *SlGRAS47*, *SlBHLH48*, and *SlMYB28*. These genes are key regulators of physiological and developmental processes. However, their roles in Pi metabolism had not been studied previously.

Using CRISPR-Cas9 gene editing, the authors generated mutants for each of these transcription factors and found that the 3 genes strongly affected plant growth, as the mutants showed lower shoot and root biomass, decreased response to Pi limitation, and lower anthocyanin accumulation. Electrophoretic mobility shift assays and chromatin immunoprecipitation assays showed that SlPHR3 and SlPHR4 bind to the promoters of these 3 genes in vitro and in vivo. These results highlight the essential roles of *SlGRAS47*, *SlBHLH48*, and *SlMYB28* in mediating the transcriptional response to phosphate limitation in tomato.

However, analysis of Pi and total P contents in roots and shoots revealed distinct differences among the mutants. Loss of *SlMYB28*, in particular, resulted in reduced Pi and total P content in both roots and shoots across all Pi conditions, suggesting that it plays a broad role in regulating phosphate homeostasis systemically throughout the plant (Fig.). Furthermore, *SlMYB28* was confirmed as a key gene in the SlPHR3 and SlPHR4 regulatory cascade. Suppression of *SlMYB28* in *SlPHR3* and *SlPHR4*-overexpressing plants reduced Pi and total P accumulation, biomass, and anthocyanin production across various Pi regimes. Moreover, expression analysis showed that *SlMYB28* regulates a suite of genes involved in Pi transport, hormone signaling, and anthocyanin biosynthesis, underscoring its integrative role in translating upstream PHR signals into physiological outcomes.

**Figure.**
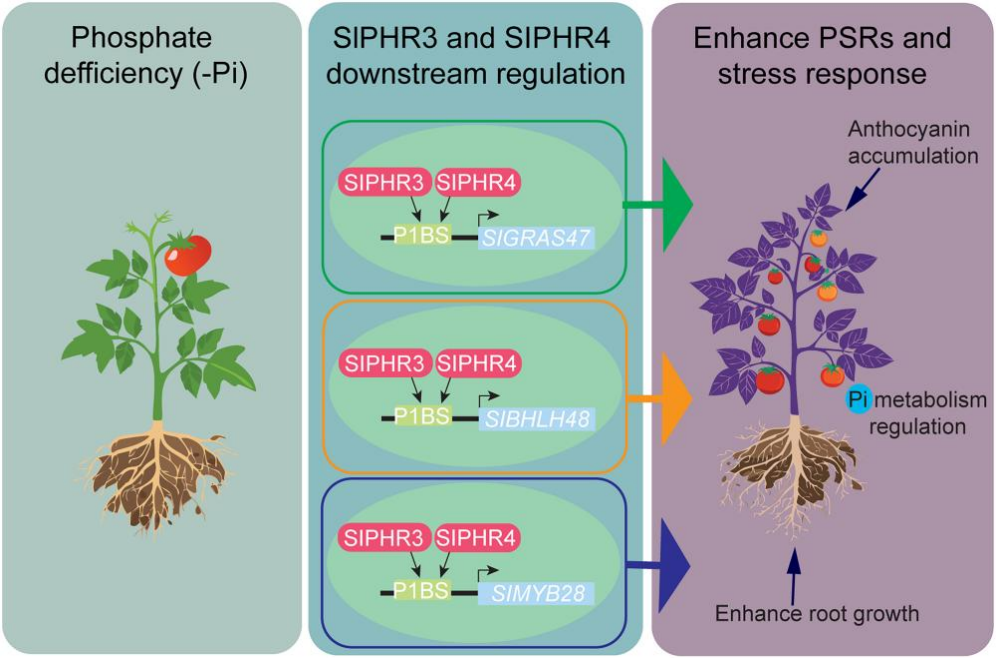
Transcriptional regulation of PSRs by SlPHR3 and SlPHR4 in tomato. Under phosphate deficiency (−Pi), tomato plants activate PSRs mediated by the transcription factors SlPHR3 and SlPHR4. These factors directly bind to the P1BS motif in the promoters of downstream transcription factor genes including *SlGRAS47*, *SlBHLH48*, and *SlMYB28*, activating their expression. These secondary transcription factors regulate processes such as root development, Pi metabolism, and anthocyanin accumulation. The resulting transcriptional cascade enhances overall PSRs and stress responses, leading to improved plant growth and nutrient use efficiency under low-Pi conditions.

In conclusion, this study reveals a transcriptional network underlying PSRs in tomato. SlPHR3 and SlPHR4 act as central regulators of Pi homeostasis, controlling root and shoot responses through direct and indirect gene regulation. The identification of downstream transcription factors such as SlGRAS47, SlBHLH48, and SlMYB28 provides a hierarchical view of the regulatory landscape governing PSRs. These findings provide new insights into the molecular mechanisms of Pi signaling in tomato and lay the groundwork for improving phosphorus-use efficiency in crop breeding.

## Recent related articles in *The Plant Cell*

- Yang et al. (2024) reviewed molecular mechanisms that regulate P starvation responses, emphasizing P transport, sensing, and signaling.
- Liao et al. (2022) uncovered a Pi-sensing module that coordinates the AM symbiosis under different Pi availability conditions.

## Dive Curated Terms

The following phenotypic, genotypic, and functional terms are of significance to the work described in this paper:

anthocyanin content Planteome: TO:0000071

PHR1 Gramene: AT4G28610
PHR1 Araport: AT4G28610
